# Targeting CRHR1 Signaling in Experimental Infantile Epileptic Spasms Syndrome: Evidence for Route-Dependent Efficacy

**DOI:** 10.3390/children13010125

**Published:** 2026-01-14

**Authors:** Tamar Chachua, Mi-Sun Yum, Chian-Ru Chern, Kayla Vieira, Jana Velíšková, Libor Velíšek

**Affiliations:** 1 Department of Cell Biology & Anatomy, New York Medical College, Valhalla, NY 10595, USA; tamarchachua@gmail.com (T.C.); chianru_chern@nymc.edu (C.-R.C.); kvieira2@student.nymc.edu (K.V.); 2 Department of Pediatrics, University of Ulsan College of Medicine, Seoul 05505, Republic of Korea; yummisun@amc.seoul.kr; 3 Department of Pediatrics, Asan Medical Center Children’s Hospital, Seoul 05505, Republic of Korea; 4 Departments of Cell Biology & Anatomy, Obstetrics and Gynecology, and Neurology, New York Medical College, Valhalla, NY 10595, USA; jana_veliskova@nymc.edu; 5 Departments of Cell Biology & Anatomy, Pediatrics, and Neurology, New York Medical College, Valhalla, NY 10595, USA

**Keywords:** intracerebroventricular, SN003, CP376395, corticotropin releasing hormone, systemic administration, hypothalamus

## Abstract

**Highlights:**

**What are the main findings?**

- CRHR1 antagonists suppress experimental infantile spasms in a route-, drug-, and brain-site-specific manner.
- Systemic SN003 reduces spasms, whereas systemic CP376395 paradoxically exacerbates them, despite both being effective when delivered intracranially.

**What are the implications of the main findings?**

- Discrete hypothalamic circuits, particularly the arcuate nucleus, play a pivotal role in spasm generation and are viable therapeutic targets.
- SN003 may be a better option for further translational development than CP376395 for CRHR1-targeted therapy in infantile epileptic spasms syndrome.

**Abstract:**

Background/Objectives: Infantile epileptic spasms syndrome (IESS) is a severe epilepsy of infancy. Corticotropin (ACTH) and vigabatrin are the only FDA-approved therapies. The efficacy of ACTH together with the strong convulsant effects of corticotropin-releasing hormone (CRH) suggests that excess CRH, secondary to impaired ACTH feedback, may contribute to spasms. We therefore hypothesized that CRH receptor 1 (CRHR1) antagonists would suppress spasms in a route- and drug-dependent manner. Methods: Using our validated rat model of IESS, in which prenatal priming with betamethasone was followed by postnatal triggering of spasms with N-methyl-D-aspartic acid (NMDA), we tested two CRHR1 antagonists, CP376395 and SN003, delivered intracranially (via intracerebroventricular or intraparenchymal infusion) or systemically. Results: Intracerebroventricular infusion of both antagonists suppressed spasms, with CP376395 providing more consistent effects. Intraparenchymal administration into the hypothalamic arcuate nucleus also reduced spasms, whereas misses into the mammillary bodies were ineffective, highlighting site specificity. Systemic administration yielded divergent results: SN003 robustly suppressed spasms, whereas CP376395 unexpectedly exacerbated them. No sex differences were observed. Conclusions: These findings demonstrate that CRHR1 blockade modifies experimental spasms in a route- and drug-specific manner and implicates discrete hypothalamic circuits, particularly those including the arcuate nucleus, in spasm generation. The divergent systemic responses between CP376395 and SN003 likely reflect differences in CRHR1 engagement (competitive and non-competitive antagonism, respectively) as well as differences in binding properties that may include differential network interactions beyond local CRH signaling or duration of receptor occupancy. In conclusion, SN003 may be a better option than CP376395 for further development as a CRHR1-targeted therapy pending additional pharmacokinetic/pharmacodynamic studies. Further work should explore dosing paradigms of CP376395 to determine if a therapeutic range for CP376395 exists.

## 1. Introduction

Soon after characterization of the amino-acid sequence of ovine corticotropin-releasing hormone (here CRH, also CRF) [1], the convulsant effects of very low doses of CRH [administered intracerebroventricularly (i.c.v.)] were described in rats. These effects included EEG spike and wave discharges originating in the amygdala and behavioral seizures with forelimb clonus, rearing, and falling, consistent with Racine scale grade 5 amygdala-kindled seizures [2,3]. This convulsant model has also been reproduced in developing rats [4]. Interestingly, the range of CRH concentrations needed in infant rats was 7.5–600 pM [4] compared to the nanomolar range required to trigger seizures in adult rats (1.5–3.75 nM [2]). The prominent convulsant effects of CRH in the immature brain [4], the decreased endogenous adrenocorticotropic hormone (ACTH) in the CSF of children with infantile epileptic spasms syndrome (IESS, previously also infantile spasms) [5], and the marked efficacy of ACTH in IESS [6,7] that persists in subjects after adrenalectomy [8,9] or after pharmacological ablation of adrenals, [10] led to a new theory. This “CRH excess theory” posited that the pathophysiology of IESS is related to an increase in endogenous CRH due to decreased endogenous ACTH feedback on CRH production; this CRH surplus then stimulates CRH-1 receptors (CRHR1) [11]. Deficient endogenous ACTH feedback, together with the corresponding CRH excess, may precipitate spasms. Therefore, using a CRH antagonist could offer a more targeted approach for IESS by directly blocking the pro-convulsant effects of CRH without the broad systemic actions of ACTH and corticosteroids. This approach might mitigate common adverse effects associated with these treatments, such as immunosuppression and metabolic disturbances, while still subduing seizure activity.

Our laboratory has developed a rat model of IESS consisting of prenatal priming with betamethasone and postnatal induction of spasms with N-methyl-D-aspartic acid (NMDA) [12]. This model exhibits semiological similarity to human spasms, recapitulates characteristic EEG signatures including ictal decrements and interictal chaotic waveforms, and responds to ACTH, vigabatrin, and corticosteroids [13]. The model has been independently reproduced in different laboratories [14,15,16,17] and provides useful insights into the pathogenesis of spasms and putative treatments [18,19]. Our original study using [^14^C]-deoxyglucose uptake and c-fos early gene immunohistochemistry identified several hypothalamic structures associated with spasms, including early activation of the hypothalamic arcuate nucleus [12].

Therefore, we employed our rat model of IESS to evaluate the anticonvulsant efficacy of CRHR1 antagonists against spasms. We selected two different CRHR1 antagonists: CP376395 and SN003. CP376395 behaves as a classical competitive antagonist with concentration-dependent effects that are strongly influenced by stress history and circuit engagement [20,21]. In contrast, SN003 exhibits insurmountable, non-competitive antagonism with slow receptor dissociation, producing a sustained CRHR1 blockade that may outlast its exposure in the brain [22,23]. Brain penetrance of both drugs has been demonstrated [20,24]. While none of the drugs have been applied in epilepsy research, both were used in stress-, anxiety-, autonomic-, and addiction-related preclinical studies. Such studies have informed our dosing strategies in the present experiments [20,22,23,25,26,27].

We hypothesized that CRHR1 blockade would suppress NMDA-triggered spasms in a route- and drug-dependent manner. In addition to administering two different antagonists, we evaluated potential route-dependent effects through systemic, intracerebroventricular (i.c.v.), and intraparenchymal administration. Our results indicate that the effects of two mechanistically distinct CRHR1 antagonists in the rat model of IESS are drug-dependent and specific to the route of administration. The paradoxical increase in the number of spasms due to systemic CP376395 may be due to engagement of different circuitry compared to SN003, or possibly from a CRH rebound effect.

## 2. Materials and Methods

### 2.1. Animals

All animal experiments were approved by the IACUC of the New York Medical College and complied with the NIH Guide for the Care and Use of Laboratory Animals, 8th edition. Timed pregnant Sprague-Dawley rats were purchased from Taconic Biosciences (Germantown, NY, USA). and delivered to the institutional Animal Facility on day 8 of pregnancy (G8). They were housed individually on a regular 12 h light/12 h dark cycle (lights on at 07:00 h) with free access to rat chow and tap water. After one week of acclimation, on G15, pregnant females were injected with betamethasone (betamethasone phosphate, MilliporeSigma, St. Louis, MO, USA, in normal saline, 2 × 0.4 mg/kg intraperitoneally = i.p.) at 08:00 and 18:00 h [12].

Delivery of the offspring was consistent on G23, equaling postnatal day 0 (P0). On P1, all newborns were sexed, marked, and the litters were culled to a maximum of 12 pups, consisting of an equal share of males and females if possible.

### 2.2. Drugs and Delivery

To determine the role of CRHR1, we used two commercially available drugs:

(1) CP376395 hydrochloride (#3212, Tocris/biotechne, Ellisville, MO, USA), which is a brain-penetrant, potent, competitive, and selective CRHR1 antagonist [20].
(2) SN003 (then #3294, Tocris/biotechne), which is a non-simple CRH antagonist at the CRHR1 [22,23].

Both antagonists were administered intracranially (i.c.v. and intraparenchymal) and systemically. CP376395 was diluted in normal saline. SN003 was first diluted in 100% ethanol, and further diluted with distilled water to create a 1% ethanol solution. Effective doses were determined in pilot experiments.

### 2.3. Surgeries

On P13, rat pups (both males and females) were anesthetized by isoflurane inhalation (induction: 5% in oxygen in the induction chamber; maintenance: 3% in oxygen through the stereotaxic instrument mask) and fixed to the stereotaxic instrument (David Kopf Instruments, Tujunga, CA, USA). The skin from the skull was removed, and the fascia was cleaned. Surface bleeding was stopped with 3% hydrogen peroxide. A position for the microinfusion guide cannula (Plastics One, now Protech International, Inc., Boerne, TX, USA) was set according to the anteroposterior and lateral coordinates (Table 1, with bregma set to zero). A small hole was drilled into the bone, and the guide cannula was lowered to the set depth. The guide cannula coordinates were set 1 mm shallower than the target structure coordinates, since the microinfusion cannula extended 1 mm beyond the guide cannula. A single guide cannula was implanted into either the lateral ventricle, the third ventricle, or the arcuate nucleus. There is extensive communication between the lateral ventricles through the interventricular foramina, justifying unilateral microinfusion [28]. After insertion of the guide cannula, two anchoring jeweler screws were inserted into the nasal and occipital bones, and the cannula–screw complex was secured by dental acrylic. The entire procedure lasted < 20 min per animal. After surgery, the animals were placed on a heated pad until ambulatory, and then reunited with their respective dams [29].

### 2.4. Intracranial Drug Microinfusions

On P15, the animals were gently immobilized in hand, and the microinfusion cannula was carefully inserted through the implanted guide cannula to the structure of interest (lateral ventricle, third ventricle, or arcuate nucleus). Solutions were microinfused using a 5 µL Hamilton microsyringe (Reno, NV, USA) attached to a Teflon tube. Drug solutions at varying concentrations (Table 1) were prepared in a fixed total volume of 0.5 µL and were slowly microinfused over 1 min; vehicle controls received 0.5 µL of vehicle over 1 min. After microinfusing, the cannula stayed in place for an additional 1 min before being retracted to prevent backflow of the solution [29]. Both CP376395 and SN003 microinfusions were completed 1 h prior to NMDA induction of spasms.

### 2.5. Systemic Drug Administration

On P15, both drugs were injected i.p. 1 h prior to the NMDA induction of spasms. CP376395 was diluted in normal saline and injected at 3 mg/kg, whereas SN003 was first diluted in 100% ethanol, then diluted with distilled water to a 1% ethanol solution and injected at 0.7 mg/kg. Controls always received equivalent volumes of respective vehicles. Drug doses were derived from published doses used for in vivo experiments in adult rats, namely, systemic CP376395 of 5.0–10.0 mg/kg [27] and systemic SN003 of 0.5–1.0 mg/kg [30].

### 2.6. NMDA Trigger of Spasms

NMDA was administered i.p. on P15 at a dose of 15 mg/kg dissolved in normal saline. Immediately after the i.p. injection, the animals were placed in separate cages on a heating pad and observed for symptomatology of NMDA syndrome. Besides marking latency to onset of tail twisting (snake-like tail movements starting at the tip), arching, and flexion spasms (emprosthotonus, hyperflexion position on the side lasting < 10 s), we also counted the total number of spasms within the 90 min observation period. Our primary outcome was the number of spasms, with a secondary outcome of latency to onset of spasms from the time point of NMDA injection.

### 2.7. Quality Control, Histology, and Statistics

All experimenters were blinded to whether animals received drug versus vehicle microinfusion. Every animal group in the main experiments was combined from at least 3 litters, and no more than 2 males and 2 females from a single litter were used. The animals were assigned to the groups using stratified randomization, i.e., randomization that accounted for the litter origin and sex. We also monitored the animals’ body weights as a simple measure of even group randomization, and there was no difference in the body weight distribution among the groups. After the experiments, all animals with intracranial cannulas were euthanized under ketamine/xylazine (7/70 mg/kg i.p.) anesthesia and brains were extracted. Brains were flash frozen, sectioned using a cryostat, and stained with cresyl violet (Nissl stain) for microscopic verification of the tip of the cannula placement. Only animals with tips in the regions of interest (lateral ventricle, third ventricle, and arcuate nucleus or mammillary body) were included in the data analysis.

In NMDA-induced spasms, we used the presence of any preceding symptoms (tail twisting, arching) as confirmation that the NMDA was successfully administered. If the animal did not exhibit any NMDA symptoms, it was excluded from further analysis.

Two statistical packages were used: StatView 5.0 (SAS, Cary, NC, USA) and SigmaStat 4.0 (Jandel Scientific, now Grafiti LLC, Palo Alto, CA, USA). All data were tested for normality (Shapiro–Wilk test) and equal variances (Brown–Forsythe test). If conditions of parametric statistics were met, data were analyzed using Student’s *t*-test (two groups) or ANOVA (multiple groups). If parametric statistics were rejected, data were analyzed using the Mann–Whitney test (two groups) or the Kruskal–Wallis test (multiple groups). Post hoc pair-wise comparisons were conducted using Bonferroni–Dunn or Dunn’s test (vs. control group) with *p* levels trimmed for multiple comparisons. The level of significance was preset to *p* < 0.05. Data presentation reflects the statistical approach used. If a parametric analysis was used, the mean and the standard error of the mean (±S.E.M.) are shown. If nonparametric evaluation was used, individual data points and the median are presented. We also tested sex as a biological variable: there was no effect of sex on the latency to onset of spasms, the number of spasms, or on drug effects, so both sexes were combined.

## 3. Results

### 3.1. Intracerebroventricular Microinfusions of the CP376395

**Figure 1:**
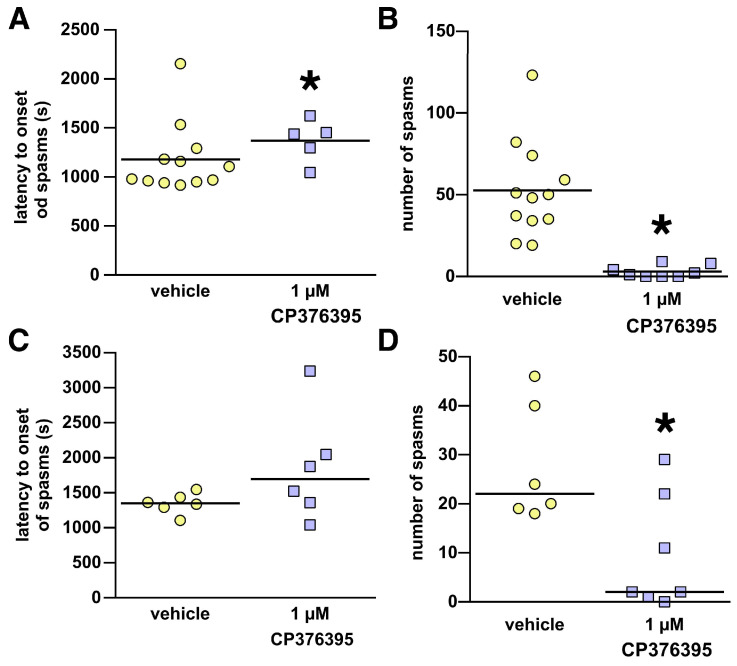
Effects of intracerebroventricular (i.c.v) administration of CP376395. Data shown here were collected from animals with a histologically verified position of the cannula tip either within the lateral (**A**,**B**) or third (**C**,**D**) ventricle. Data presentation follows statistical evaluation: if the data conform to parametric statistical inference, the data mean ± S.E.M. are shown. If nonparametric testing was used, individual data points and the data median (black line) are shown. (**A**,**B**) Lateral ventricle administration of 1 µM CP376395. (**A**) CP significantly delayed the onset of spasms; * *p* < 0.05. The difference between the number of data points in A and B illustrates the complete suppression of spasms in some subjects. (**B**) Significant suppression of spasms by CP376395 in comparison with vehicle-infused controls; * *p* < 0.05. (**C**,**D**) Study with third ventricle administration of 1 µM CP376395. (**C**) No difference in latency to onset of spasms between the groups. (**D**) Significant suppression of spasms by 1 µM CP376395 compared to vehicle, * *p* < 0.05.

Pilot experiments indicated that a 1 µM concentration of CP376395 was effective. Microinfusion of this concentration of CP376395 into the lateral ventricle significantly delayed the latency to onset of spasms (Mann–Whitney U = 13.00; tied * *p* = 0.0142; Figure 1A) and profoundly reduced the number of spasms (Mann–Whitney U = 0.000; tied * *p* = 0.0009; Figure 1B).

To further explore the effects of CP376395, we tested 1 µM CP376395 in the third ventricle. There was no effect of 1 µM CP376395 infusion into the third ventricle on the latency to onset of spasms (Mann–Whitney U = 10.000; tied *p* = 0.2002; Figure 1C). However, microinfusion of CP376395 in the third ventricle significantly suppressed the number of spasms (Mann–Whitney U = 7.000; tied * *p* = 0.0452; Figure 1D).

### 3.2. Targeted Brain Intraparenchymal Microinfusions of the CP376395

**Figure 2:**
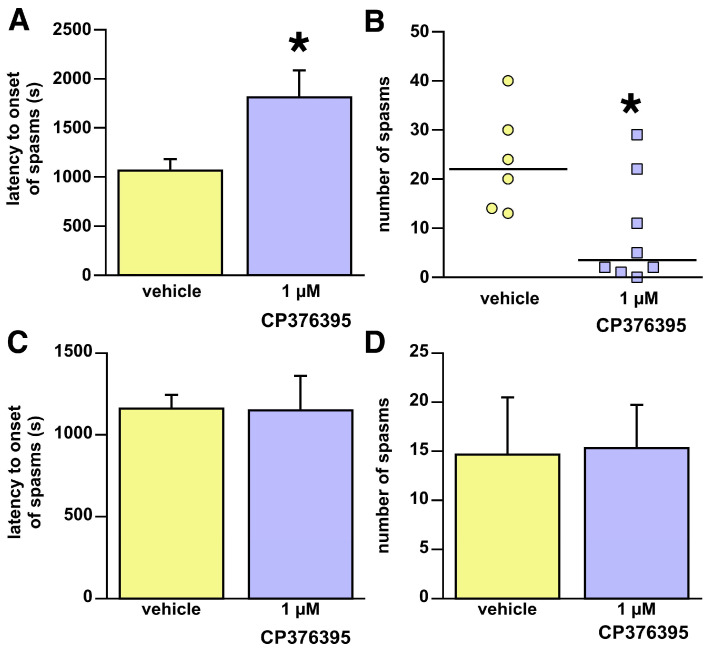
Effects of brain intraparenchymal administration of CP376395. Data shown here were collected from animals with a histologically verified position of the cannula tip either within the intended target of the hypothalamic arcuate nucleus (**A**,**B**) or misses within the mamillary bodies (**C**,**D**). Data presentation follows statistical evaluation (see Figure 1 for details). (**A**,**B**) Intraparenchymal administration of CP376395 into the hypothalamic arcuate nucleus. (**A**) CP376395 significantly delayed the onset of spasms compared to the vehicle; * *p* < 0.05. (**B**) CP376395 significantly suppressed the occurrence of spasms compared to vehicle; * *p* < 0.05. (**C**,**D**) Microinfusions of CP376395 into the mamillary body (misses from the arcuate nucleus microinfusions). (**C**) No effect on the latency to onset of spasms. (**D**) No effect on the number of spasms per observation period. The results indicate that in the brain, CP376395 has site-specific effects on spasms; * *p* < 0.05.

Our previous data indicated that the hypothalamic arcuate nucleus was significantly involved in the expression of spasms [12]. CRHR1 is highly expressed in the arcuate nucleus [31,32]. Hence, we were interested in whether direct microinfusions of CP376395 into the arcuate nucleus would influence spasms. Notably, 1 µM CP376395 delayed the latency to onset of spasms (Student’s *t*-test; t = 2.365; 11df; * *p* = 0.0375; Figure 2A) and suppressed the number of spasms (Mann–Whitney U = 7.000; tied * *p* = 0.0280; Figure 2B). To assess site specificity, we analyzed off-target microinfusions (misses) that ended in the mamillary bodies, which have lower CRHR1 expression [32] and are not implicated in NMDA-induced spasms [12]. There was no effect of CP376395 microinfusions (1 µM group n = 4; 1 mM group n = 3; control group n = 3) on the latency to onset of spasms (Student’s *t*-test t = −0.37; 7df; *p* = 0.9718; Figure 2C) or number of spasms (Student’s *t*-test t = 0.089; 7df; *p* = 0.9312; Figure 2D). While the power of this experiment is low, a post hoc sample size analysis determined that given the current outcome, the effect size is 0.091, requiring a sample size of 386 per subgroup to achieve significant difference at *p* = 0.05 and power (1 − β) = 0.8.

### 3.3. Intracerebroventricular Microinfusions of the SN003

**Figure 3:**
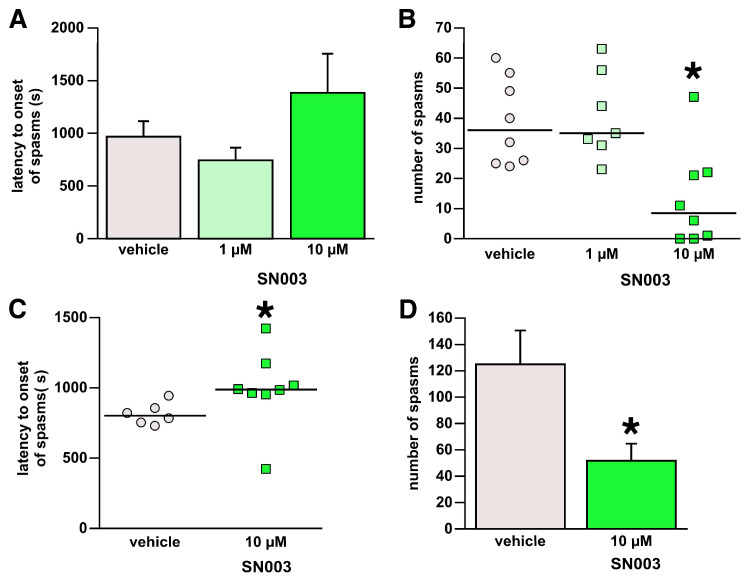
Effects of intracranial administration of SN003. Data shown here were collected from animals with a histologically verified position of the cannula tip either within the lateral ventricle (**A**,**B**) or the hypothalamic arcuate nucleus (**C**,**D**). Data presentation follows statistical evaluation (see Figure 1 for details). (**A**,**B**) Intracerebroventricular administration of SN003 in the lateral ventricle. (**A**) There were no effects of the two SN003 concentrations on the latency to onset of spasms. (**B**) Only the higher 10 µM concentration of SN003 (volume of 0.5 µL) significantly suppressed the occurrence of spasms; * *p* < 0.05. (**C**,**D**) Intraparenchymal administration of SN003 (10 µM) in the hypothalamic arcuate nucleus. (**C**) SN003 significantly delayed the onset of spasms in comparison to the vehicle-microinfused group; * *p* < 0.05. (**D**) SN003 significantly suppressed the number of spasms; * *p* < 0.05.

We tested two concentrations of SN003 microinfused into the lateral ventricle: 1 µM and 10 µM. There was no effect of SN003 microinfusions on the latency to onset of spasms (ANOVA F(2,18) = 2.032; *p* = 0.160; Figure 3A). However, we found a significant effect of the SN003 microinfusion on spasms (Kruskal–Wallis H = 10.463; 2df; *p* = 0.005). Specifically, the higher concentration of SN003 (10 µM) significantly suppressed spasms in comparison to controls (Dunn’s test; * *p* = 0.013; Figure 3B), consistent with the effect of the other CRHR1 antagonist, CP376395.

### 3.4. Targeted Brain Intraparenchymal Microinfusions of the SN003

For microinfusions into the arcuate nucleus, we used the effective concentration of SN003 (10 µM). We observed the 10 µM concentration of SN003 had the effect of increasing the latency to onset of spasms (Mann–Whitney U = 6.000; * *p* = 0.020; Figure 3C). Similarly, SN003 decreased the number of spasms (Student’s *t*-test; t = 2.731; 12df; *p* = 0.018; Figure 3D).

### 3.5. Systemic Administration of CP376395

**Figure 4:**
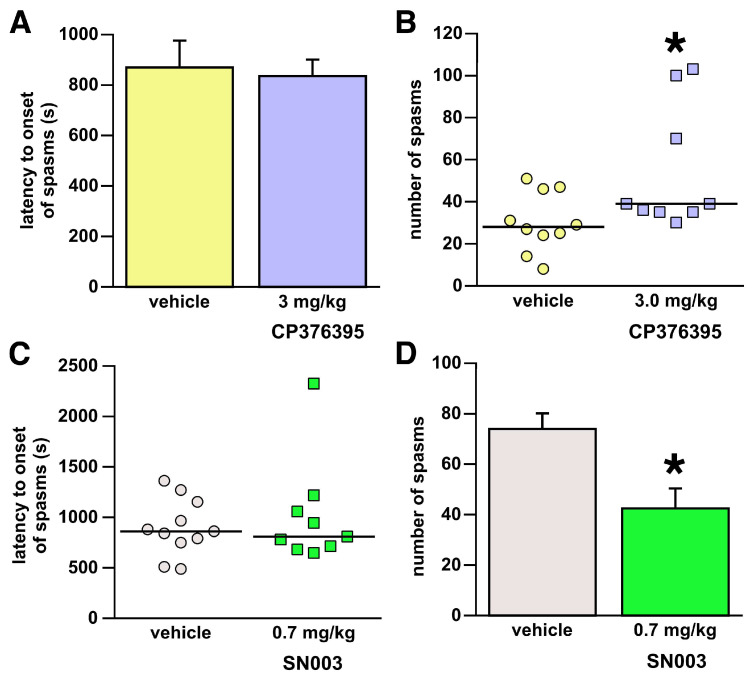
Effects of systemic administration of CRHR1 antagonists. Both CP376395 and SN003 were administered i.p. one hour prior to the NMDA induction of spasms. Data presentation follows statistical evaluation (see Figure 1 for details). (**A**,**B**) Intraperitoneal administration of 3 mg/kg of CP376395. (**A**) There was no effect of CP376395 on the latency to onset of spasms. (**B**) CP376395 significantly increased the occurrence of spasms; * *p* < 0.05. (**C**,**D**) Intraperitoneal administration of 0.7 mg/kg of SN003. (**C**) There was no effect of SN003 on the latency to onset of spasms. (**D**) SN003 significantly suppressed the occurrence of spasms; * *p* < 0.05.

There were no differences in latency to onset of spasms (Student’s *t*-test, t = −0.262; 17df; *p* = 0.7967; Figure 4A). To our surprise, systemic administration of CP376395 increased the number of spasms: Mann–Whitey U = 19.000 and tied * *p* = 0.0336, as shown in Figure 4B; CP376395 median = 39.0; 25th–75th percentile = 35.0–85.0; and vehicle median = 28.0 (21.5–46.25).

### 3.6. Systemic Administration of SN003

SN003 did not alter latency to onset of spasms (Mann–Whitney U = 48.000; tied *p* = 0.9093; Figure 4C). However, i.p. administration of SN003 significantly decreased the number of spasms in comparison to the vehicle group (Student’s *t*-test; t = 3.145; 18df; * *p* = 0.0056; Figure 4D).

## 4. Discussion

In summary, the effects of the two CRHR1 antagonists, SN003 and CP376359, on spasm burden were route-, agent-, and site-specific, likely reflecting their distinct features. Intracranial administration of CP376395 produced broad and consistent effects, generally increasing the latency to spasms and suppressing their occurrence. Intracranial delivery of SN003, by contrast, was more effective at increasing latency rather than reducing the number of spasms. Interestingly, after systemic intraperitoneal administration, CP376395 exacerbated spasms, while SN003 suppressed them. We showed here that CRHR1 receptors participate in the development of spasms in the rat model of IESS. However, we did not confirm their consistent contribution as expected, suggesting that not every CRHR1 antagonist can serve as an efficacious therapeutic intervention for IESS.

We observed distinct site-specific actions. Both antagonists were effective when infused into the lateral ventricle as well as into the hypothalamic arcuate nucleus. These findings are consistent with our prior findings of the arcuate nucleus’ involvement in spasms [12], and with the known high concentration of CRHR1 in this region [31,33]. The absence of an effect in the mammillary bodies after CP376395 infusions further supports a selective role of specific hypothalamic circuits in the pathophysiology of spasms. Our endpoints were the number of spasms per 90 min observation period (primary) and the latency to onset of spasms (secondary). The spasms observed in our model were overwhelmingly flexion spasms, which usually cluster under control conditions when a sufficient number of spasms develop [34]. The spasms lasted only a few seconds (akin to spasms in children). However, under certain conditions (not employed in this study) NMDA-induced spasms may convert to long-lasting spasms, which can span 30 s to several minutes. Besides the features of the NMDA-induced spasm syndrome, no other behavioral changes were observed in vehicle controls or drug-treated animals.

Differences between the concentrations of antagonists required for the effects may reflect differences in their inherent features. Intracranially, CP376395 was slightly more potent than SN003, indicating that CP376395 may be more effective at preventing local triggering. CP376395 is a CRHR1 antagonist with relatively short plasma half-life; fast kinetics; and largely competitive, concentration-dependent features [20,27,35]. Thus, after local administration, its pharmacodynamic profile is consistent with concentration-dependent receptor blockade. Unlike CP376395, SN003 does not conform to simple competitive antagonism at the CRHR1 [22,23]. Its kinetics at the CRHR1 receptor are slower and thus do not reflect plasma concentrations [22,36]. There was also a difference in the effects between the microinfusion sites (lateral vs. third ventricle). While this may have reflected potential off-target effects, we were unable to find support for this in the literature. Another explanation is differential involvement of adjacent structures, depending on the drug concentration and the circuitry of those structures. Finally, signal to noise (due to the small cohort) may also have contributed to the differential effects.

Spasms in this model are unlikely to be explained solely by a CRH-driven mechanism, despite its attractive plausibility. In the immature brain, excess CRH triggers seizures that originate in the amygdala, which develop into a specific phenotype characterized by clonic forelimb seizures with preserved righting [4,37]. These seizures are phenotypically different from the NMDA-induced spasms seen in infant rats [12]. This significantly limits a simplistic “CRH-only” explanation. A more relevant interpretation is that NMDA (or glutamate, possibly in combination with prenatal betamethasone exposure) promotes CRH release within hypothalamic nuclei [38]. This contributes to the triggering of spasms in structures distinct from the medial amygdala and enhances brainstem excitability required to sustain the spasm phenotype [12]. We have also observed a dissociation between the effects on seizure latency to onset and the number of spasms. Delayed initiation of spasms by the CRHR1 antagonists (as evidenced by increased latency to onset of spasms) may be due to the increased thresholds at trigger sites, such as the arcuate nucleus. Indeed, there may be dissociation between the activity of the trigger site and the circuitry needed for the expression of spasms, which may include brain stem structures [12,39,40]. Based on the receptor distribution, drug activity, and possible off-target interaction, any dissociation of the latency and the number of spasms may be observed.

Intracranial infusions produced relatively consistent effects with both antagonists. However, SN003 and CP376395 diverge in vivo because SN003 frequently behaves as a non-simple CRHR1 antagonist, exhibiting incomplete surmountability and apparent reductions in agonist binding capacity in some assay contexts [36]. This behavior is accompanied by moderately persistent receptor engagement, with dissociation kinetics on the order of tens of minutes to approximately one hour [36], which can yield effects that modestly outlast peak systemic exposure. Importantly, SN003 has demonstrated clear in vivo CNS activity under conditions of elevated HPA/CRH tone, reducing CRH levels in stress-responsive brain regions and potentiating antidepressant-like effects in corticosterone-treated rats [41]. In contrast, CP376395 emerges from a discovery program emphasizing potent, selective, and largely competitive CRHR1 antagonism with CNS penetration inferred from ex vivo cortical receptor occupancy following systemic dosing [20]. Accordingly, CP376395 displays strong circuit- and state-dependence in vivo, with efficacy most evident in paradigms that recruit CRHR1 signaling through repeated stress or intermittent challenge, and with marked regional specificity across cortical and hypothalamic circuits. These compound-specific patterns are consistent with the broader CRHR1 antagonist literature, indicating that behavioral and physiological efficacy is greatest when endogenous CRH systems are actively engaged rather than at basal tone [21,27]. Another speculative explanation for the increased number of spasms after systemic CP376395 may be based on its short receptor occupancy: a CRH rebound within the observation period may contribute to the increased number of spasms.

Limitations of this study include the absence of higher systemic doses of CP376395, the lack of repeated systemic administration paradigms as per our IESS model [19], and the use of different vehicles for the two compounds, one with 1% alcohol. We did not assess receptor occupancy or downstream signaling measures to confirm pharmacodynamic engagement at infusion sites. Thus, the study may have benefited from a range of pre-treatment intervals. In the context of statistical evaluation, one should be aware that repeated comparison increases the chance of positive findings, though for multiple comparisons within one experimental group, we always trimmed the *p* level of the post hoc tests accordingly.

## 5. Conclusions

In conclusion, SN003 may be a better option for translational development than CP376395. However, further work is needed. This should include investigation of the drugs in different IESS models, exploring different doses and treatment paradigms including pharmacokinetics/pharmacodynamics, concordant changes in EEG activity, and the involvement of specific hypothalamic nuclei.

## Figures and Tables

**Table 1:** Doses and targets for intracranial microinfusions of CRHR1 antagonists.

Target Structure—Route (Coordinates) ^1^	DRUG	
	CP376395 Vehicle = Normal Saline	SN003 Vehicle = 1% Alcohol in Normal Saline
Third ventricle—icv (−2.0; 2.1; 6.0)	0.5 µL of 1 µM or 1 mM solution	N/A
Lateral ventricle—icv (−0.9; 1.3; 3.0)	0.5 µL of 1 µM solution	0.5 µL of 1 or 10 µM solution
Hypothalamic arcuate nucleus—intraparenchymal (−2.4; 2.3; 7.5)	0.5 µL of 1 µM solution	0.5 µL of 10 µM solution

^1^ Coordinates for intracranial cannulas are in mm (anteroposterior from bregma; lateral from sagittal suture; depth from the skull surface) with cannula inclination from the sagittal plane of 15 degrees to avoid the superior sagittal sinus. icv = intracerebroventricularly; N/A = not applicable. Arcuate nucleus microinfusion misses with confirmed positioning in the mamillary bodies served as a control for site specificity.

## Data Availability

Data will be available in PubMed Central.

## Abbreviations

The following abbreviations are used in this manuscript:

ACTH	Adrenocorticotropic hormone, corticotropin
CRH	Corticotropin-releasing hormone
CRHR1	Corticotropin-releasing hormone receptor subtype 1
IACUC	Institutional Animal Care and Use Committee
IESS	Infantile epileptic spasms syndrome
NMDA	N-methyl-D-aspartic acid
